# Erectile Dysfunction in Diabetes Mellitus: A Comprehensive Narrative Review of Pathophysiology, Genetic Association Studies and Therapeutic Approaches

**DOI:** 10.1002/edm2.70099

**Published:** 2025-09-17

**Authors:** Boštjan Hostnik, Gašper Tonin, Andrej Janež, Jasna Klen

**Affiliations:** ^1^ Division of Internal Medicine, Department of Endocrinology, Diabetes and Metabolic Diseases University Medical Centre Ljubljana Ljubljana Slovenia; ^2^ Faculty of Medicine University of Ljubljana Ljubljana Slovenia; ^3^ Division of Surgery, Department of Abdominal Surgery University Medical Centre Ljubljana Ljubljana Slovenia

**Keywords:** diabetes mellitus, erectile dysfunction, genetic polymorphisms, International Index of Erectile Function, pathophysiology

## Abstract

**Introduction:**

Erectile dysfunction (ED) is a highly prevalent complication of diabetes mellitus (DM), significantly impairing quality of life and psychosocial well‐being. The prevalence of ED is estimated to be over 3.5 times higher in men with diabetes mellitus compared to those without. The aetiology of diabetic ED is multifactorial, stemming from complex diabetes mellitus‐related systemic changes. The pathophysiology of diabetic ED involves interacting pathways, including endothelial dysfunction, accelerated atherosclerosis, autonomic and peripheral neuropathy, structural penile changes, hormonal imbalances, and psychological factors.

**Methods:**

A review of the literature was conducted to examine the pathophysiological mechanisms, genetic associations, and treatment modalities related to diabetic ED. Particular attention was given to studies exploring pharmacogenetics and emerging therapeutic interventions.

**Results:**

Management is multimodal, including lifestyle changes, counselling, and pharmacological agents (primarily phosphodiesterase type 5 inhibitors (PDE5Is)), but treatment response varies. Genetic studies have identified associations between ED risk/severity and polymorphisms in several candidate genes, including *NOS3* (G894T, T786C, VNTR), *ARG1/ARG2* (influencing nitric oxide substrate availability), ACE (I/D polymorphism), *AR* (CAG repeat length affecting androgen sensitivity), and *VEGF* (promoter polymorphisms). Pharmacogenetic studies suggest that polymorphisms in *NOS3*, *AR*, and *VEGF* may predict response to PDE5Is or testosterone therapy, while *ARG1/ARG2* variations might guide future arginase‐targeted therapies. Emerging treatments like low‐intensity shockwave therapy, platelet‐rich plasma, gene therapy, and stem cell therapy show promise but require more robust evidence.

**Conclusions:**

Diabetic ED is a complex condition driven by multiple pathophysiological mechanisms often influenced by an underlying genetic predisposition. Understanding the interplay between pathophysiology and genetics is crucial for developing personalised treatment strategies. While current therapies offer benefits, variability in response highlights the need for tailored approaches. Further research, especially large‐scale pharmacogenetic studies and randomised controlled trials for emerging therapies, is essential to identify reliable biomarkers, optimise treatment selection, and improve outcomes for men with diabetic ED.

## Graphical abstract

Interplay between erectile dysfunction, diabetes mellitus and genetics.

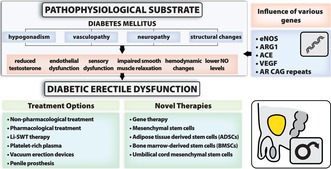

**Abbreviations**
ACE: angiotensin‐converting enzyme
ADSCs: adipose tissue‐derived stem cells
AGEs: advanced glycation end‐products
AR: androgen receptor
ARG1: arginase 1
ARG2: arginase 2
ART: androgen replacement therapy
BDNF: brain‐derived neurotrophic factor
BMSCs: bone marrow‐derived stem cells
cAMP: cyclic adenosine monophosphate
cGMP: cyclic guanosine monophosphate
CKD: chronic kidney disease
CVD: cardiovascular disease
DKA: diabetic ketoacidosis
DM: diabetes mellitus
DSPN: distal symmetric polyneuropathy
EAU: European Association of Urology
ECM: extracellular matrix
ED: erectile dysfunction
EDHF: endothelium‐derived hyperpolarising factor
EGF: epidermal growth factor
EMA: European Medicines Agency
eNOS/NOS3: endothelial nitric oxide synthase
ERK: extracellular signal‐regulated kinase
ESRD: end‐stage renal disease
ET‐1: endothelin‐1
FGF: fibroblast growth factor
fT: free testosterone
GAD65: glutamic acid decarboxylase 65
GPCRs: G‐protein‐coupled receptors
GWAS: genome‐wide association studies
HbA1c: haemoglobin A1c (Glycated haemoglobin)
HPT: hypothalamic–pituitary‐Testicular
ICI: intracavernosal injection
IGF‐1: insulin growth factor
IIEF: International Index of Erectile Function
iNOS/NOS2: inducible nitric oxide synthase
IP3: inositol triphosphate
ISSM: International Society for Sexual Medicine
LH: luteinising hormone
Li‐SWT: low‐intensity extracorporeal shock wave therapy
LUTS: lower urinary tract symptoms
MMP: matrix metalloproteinase
MMP‐9: matrix metalloproteinase 9
MSCs: mesenchymal stem cells
MUSE: medicated urethral system for erection
nNOS/NOS1: neuronal nitric oxide synthase
NO: nitric oxide
NPTR: nocturnal penile tumescence and rigidity
p38 MAPK: p38 Mitogen‐Activated Protein Kinase
PDE5: phosphodiesterase type 5
PDE5Is: phosphodiesterase type 5 inhibitors
PDGF: platelet‐derived growth factor
PGE1: prostaglandin E1
PKA: protein kinase A
PKC: protein kinase C
PKG: protein kinase G
PPI: Penile prosthesis implantation
PPP: platelet‐poor plasma
PRP: platelet‐rich plasma
PSA: prostate‐specific antigen
RAAS: Renin‐angiotensin‐aldosterone system
RCTs: Randomised Controlled Trials
ROCK: Rho‐kinase
SD: sexual dysfunction
sGC: soluble guanylate cyclase
SHBG: sex hormone‐binding globulin
SHIM: sexual health inventory for men
SNP: single nucleotide polymorphism
SNRIs: serotonin‐norepinephrine reuptake inhibitors
SSRIs: selective serotonin reuptake inhibitors
T1DM: Type 1 diabetes mellitus
T2DM: Type 2 diabetes mellitus
TCAs: tricyclic antidepressants
TGF‐β: transforming growth factor‐beta
UC‐MSCs: umbilical cord‐derived mesenchymal stem cells
VEDs: vacuum erection devices
VEGF: vascular endothelial growth factor
VNTR: variable number tandem repeat

## 1 Introduction

Erectile dysfunction (ED), a common yet often underreported complication of diabetes mellitus (DM), significantly impacts the quality of life, self‐esteem, and psychological well‐being of affected men. Defined as the persistent inability to achieve and/or maintain an erection sufficient for satisfactory sexual activity, ED affects an estimated 50% or more of men with DM [1]. Furthermore, the prevalence of ED is at least threefold higher in men with DM compared to men without the disease [2].

DM is a group of metabolic disorders characterised by impaired carbohydrate metabolism, in which glucose is underutilised as an energy source and overproduced due to dysregulation of gluconeogenesis and glycogenolysis, ultimately leading to hyperglycaemia. There are many different forms of DM, each distinguished by unique aetiologies and pathophysiological mechanisms. It remains one of the most widespread chronic diseases, with a rapidly increasing global incidence and prevalence. An estimated 537 million adults aged 20–79 years currently live with DM worldwide, contributing directly and indirectly to approximately 6.7 million deaths globally in 2021 alone. The global prevalence of DM in adults aged 20–79 years was estimated at 10.5% (standardised to the 2021 United Nations population), with the rate peaking at 24.0% in the 75–79 age group [3].

The pathophysiological link between DM and ED involves a complex interplay of vascular, hormonal, neurological, psychological and iatrogenic/lifestyle factors, ultimately disrupting the delicate neurovascular mechanisms required for normal erectile function. Although not life‐threatening, ED can be an early marker of systemic vascular damage in men with DM [4]. It negatively impacts the quality of life for affected men and their partners [5, 6] and imposes a substantial economic burden on employers due to increased absenteeism and reduced workplace productivity [6]. The condition is also associated with numerous psychosocial consequences, including reduced self‐esteem, anxiety, depression and diminished confidence and intimacy [7].

This review explores the mechanisms by which DM increases the risk of ED. Recent advances in genetics provide novel insights into ED susceptibility in patients with DM, as several polymorphisms and genetic pathways have been identified as significant contributors to the condition. Furthermore, given the expanding landscape of ED treatment options, we summarise current pharmacological and non‐pharmacological approaches, highlighting promising emerging therapies. Our goal is to facilitate the integration of personalised medicine into clinical practice by synthesising recent advancements in the understanding of risk factors, epidemiology, pathophysiology and treatment modalities, with a particular emphasis on genetic contributions.

## 2 Understanding ED in Diabetes Mellitus: Epidemiology, Pathophysiology and Treatment

A comprehensive understanding of ED in diabetes requires an examination of its epidemiological patterns, physiological basis and the unique pathophysiological changes induced by hyperglycaemia.

### 2.1 Epidemiology and Risk Factors of ED in Diabetes Mellitus

Men with DM have a significantly higher risk of ED compared to men without diabetes. A study by Corona et al. [8] reported prevalence rates of 19.4%, 15.4%, 10.4% and 21.6% for mild, mild‐to‐moderate, moderate and severe ED, respectively, in men with DM. The severity of ED is influenced by the type and duration of DM, treatment regimens and coexisting conditions [9]. Fedele et al. [10], in a study of a large population of men with DM, found a 26% prevalence of ED in men with T1DM and a 37% prevalence in men with T2DM. Similarly, a meta‐analysis by Kouidrat et al. [1] reported an overall ED prevalence of 52.5% in men with DM, with 37.5% in men with T1DM and 66.3% in men with T2DM.

The primary risk factors for ED include age, DM, dyslipidaemia, hypertension, cardiovascular disease (CVD), obesity, metabolic syndrome, hyperhomocysteinaemia, physical inactivity and smoking. ED is also linked to pharmacological treatments for CVDs. Thiazide diuretics and most beta‐blockers (with the exception of nebivolol) may increase the risk, whereas angiotensin‐converting enzyme (ACE) inhibitors, angiotensin II receptor blockers (ARBs) and calcium channel blockers have demonstrated neutral or potentially beneficial effects. Psychotropic medications, including antidepressants (selective serotonin reuptake inhibitors [SSRIs] and tricyclics) and antipsychotics, also increase ED risk. Other contributing factors include atrial fibrillation, hyperthyroidism, vitamin D deficiency, hyperuricaemia, folate deficiency, depression, anxiety, chronic kidney disease (CKD) and rheumatic diseases. ED often co‐occurs with other urological conditions, such as lower urinary tract symptoms (LUTS), benign prostatic hyperplasia (BPH), a history of prostate surgery, chronic prostatitis, chronic pelvic pain syndrome, painful bladder syndrome, premature ejaculation and surgical urethroplasty for posterior urethral strictures [11, 12, 13, 14].

### 2.2 Physiology of Erection and Detumescence

Penile erection is a complex physiological process integrating neurological, vascular and endocrine factors. It involves arterial dilation, relaxation of trabecular smooth muscle and activation of the corporeal veno‐occlusive mechanism [15].

#### 2.2.1 Hemodynamics and Mechanism of Erection and Detumescence

In the flaccid state, smooth muscle of the corpora cavernosa is tonically contracted, allowing only minimal arterial flow for nutritional needs. The intracavernosal partial pressure of oxygen (PO_2_) is approximately 35 mmHg. This flaccid state represents a moderate level of contraction, demonstrable by further contraction in response to cold or phenylephrine injection. Sexual stimulation triggers neurotransmitter release from cavernous nerve endings, initiating smooth muscle relaxation and subsequent haemodynamic events [16].

Initially, arterioles and arteries dilate, increasing blood flow during both diastole and systole. This leads to engorgement of the sinusoidal spaces with blood. Subsequently, compression of the subtunical venular plexus between the tunica albuginea and the peripheral sinusoids reduces venous outflow. Further occlusion of the emissary veins occurs as the tunica albuginea stretches to its capacity, minimising venous outflow. The resulting increase in intracavernosal PO_2_ (to approximately 90 mmHg) and pressure (to approximately 100 mmHg) elevates the penis to a fully erect state. Contraction of the ischiocavernosus muscles further increases intracavernosal pressure (to several hundred mmHg), resulting in the rigid erection phase [17].

#### 2.2.2 Molecular Mechanism of Smooth Muscle Contraction and Relaxation

Changes in cytosolic Ca^2+^ concentration regulate smooth muscle contraction and relaxation. Receptors on smooth muscle cells are activated by various neurotransmitters and vasoactive substances, such as norepinephrine (from nerve endings), endothelin‐1 (ET‐1) and prostaglandin F2α (from the endothelium). This process increases the levels of inositol triphosphate (IP3) and diacylglycerol (DAG). These molecules, in turn, raise cytosolic Ca^2+^ by promoting its release from intracellular stores (mainly the sarcoplasmic reticulum) and facilitating its influx through cell membrane channels [18].

As cytosolic Ca^2+^ levels normalise, Ca^2+^‐sensitising pathways become dominant. One involves excitatory G‐protein‐coupled receptors (GPCRs), which enhance Ca^2+^ sensitivity without altering cytosolic Ca^2+^ levels [19]. This pathway involves RhoA, a small monomeric G protein that activates Rho‐kinase. Rho‐kinase then phosphorylates and inhibits myosin light chain phosphatase (MLCP), maintaining contractile tone by preventing myosin light chain dephosphorylation. While phasic contraction is driven by increased Ca^2+^, tonic contraction is sustained by Ca^2+^‐sensitising pathways [17].

The process of smooth muscle relaxation is initiated by a reduction in sarcoplasmic free Ca^2+^. This causes calmodulin to dissociate from myosin light chain kinase (MLCK), which in turn inactivates the enzyme. With MLCK inactive, MLCP dephosphorylates the myosin light chains. This dephosphorylation results in actin detachment and muscle relaxation. Additionally, cyclic nucleotides like cAMP and cGMP promote relaxation by activating their respective kinases, PKA and PKG. These kinases phosphorylate target proteins and ion channels, resulting in K^+^ channel opening (hyperpolarisation), Ca^2+^ sequestration into the sarcoplasmic reticulum and inhibition of voltage‐gated calcium channels. These combined actions reduce cytosolic free Ca^2+^, promoting smooth muscle relaxation [17].

#### 2.2.3 Neuroanatomy and Neurophysiology of Erection and Detumescence

Penile innervation includes autonomic (sympathetic and parasympathetic) and somatic (sensory and motor) components. Sympathetic and parasympathetic fibres originate from the spinal cord and peripheral ganglia and converge in the pelvic plexus to form the cavernous nerves. These nerves innervate the corpora cavernosa and corpus spongiosum, playing a key role in regulating both erection and detumescence. Somatic nerves mediate penile sensation and control the bulbospongiosus and ischiocavernosus muscles [20]. Sympathetic fibres originate from T11 to L2 segments, pass through the sympathetic chain and reach the pelvic plexus via lumbar splanchnic and hypogastric nerves. Parasympathetic fibres arise from S2 to S4 segments and travel via pelvic nerves to synapse in the pelvic plexus, where they join sympathetic fibres [21]. The cavernous nerves, branches of this plexus, are critical for erectile function but are susceptible to injury during pelvic surgeries. Stimulation of the pelvic plexus and cavernous nerves promotes erection via sacral parasympathetic pathways, while sympathetic trunk activation induces detumescence via thoracolumbar pathways [22].

### 2.3 Pathogenesis and Pathophysiology of ED

ED can be classified in several ways. Based on aetiology, it can be categorised as iatrogenic, traumatic, or diabetic. Based on the underlying physiological dysfunction, it can be classified as neurogenic, arteriogenic, or venogenic. The International Society for Sexual Medicine (ISSM) recommends a primary distinction between organic and psychogenic ED. Organic ED is further subdivided into vasculogenic (arteriogenic, venogenic, or mixed), neurogenic, anatomic and endocrinologic causes. Psychogenic ED is classified as generalised or situational [15, 17, 23].

Neurogenic and vasculogenic factors are the most common causes of ED, becoming more prevalent with age and often co‐occurring with comorbidities such as hypertension, DM, atherosclerosis, hyperlipidaemia and metabolic syndrome [24]. Diabetic ED arises from a multifactorial pathophysiology, presenting challenges for effective risk factor modification and treatment.

### 2.4 Pathophysiology of ED in Patients With Diabetes Mellitus

The pathogenesis of diabetic ED is multifactorial, with several risk factors identified in patients with DM. A recent meta‐analysis of 66,925 patients with DM demonstrated that the following were associated with an increased risk of ED: higher HbA1c (OR: 1.44, 95% CI = 1.28–1.62), longer DM duration (OR: 1.39, 95% CI = 1.29–1.50), diabetic neuropathy (OR: 3.47, 95% CI = 2.16–5.56), diabetic retinopathy (OR: 3.01, 95% CI = 2.02–4.48), CVD (OR: 1.92, 95% CI = 1.71–2.16), microvascular disease (OR: 2.14, 95% CI = 1.61–2.85), general vascular disease (OR: 2.75, 95% CI = 2.35–3.21), nephropathy (OR: 2.67, 95% CI = 2.06–3.46) and metabolic syndrome (OR: 2.22, 95% CI = 1.98–2.49) [25]. Many of these risk factors are directly involved in the pathophysiology of DM itself. However, the majority of foundational studies exploring the pathophysiology of diabetic ED were conducted in the previous millennium. Therefore, newer studies emphasising genetic, epigenetic and molecular mechanisms are needed.

**FIGURE 1:**
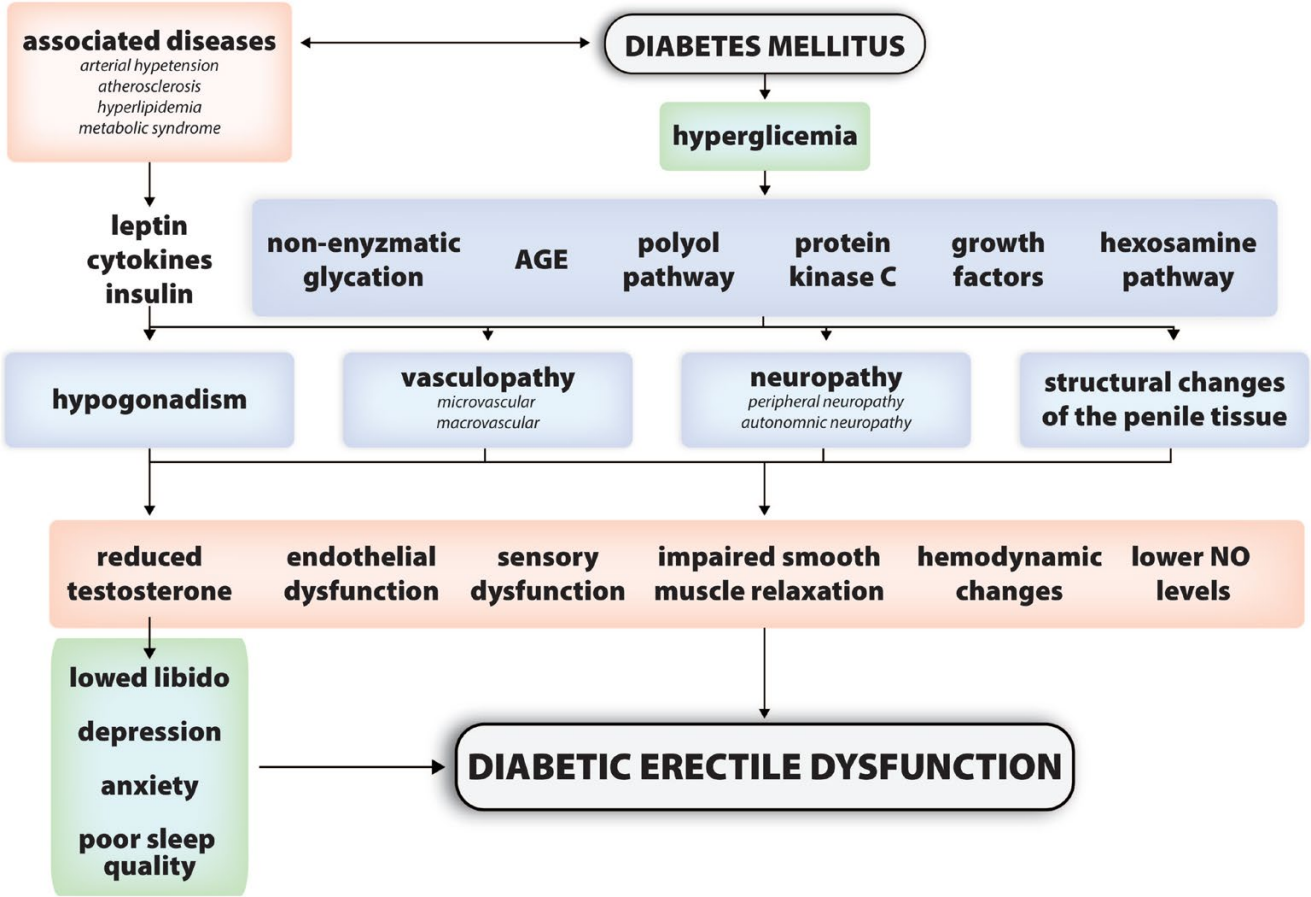
The diagram shows the multifaceted pathophysiological background of diabetic erectile dysfunction (ED) as a consequence of diabetes and related metabolic disorders. Chronic hyperglycaemia in diabetes triggers a number of biochemical pathways, including non‐enzymatic glycation, accumulation of glycation end‐products (AGEs), activation of the polyol pathway, protein kinase C, growth factors and the hexosamine pathway. These processes lead to the development of micro‐ and macrovasculopathy, peripheral and autonomic neuropathy, structural changes in penile tissue and hormonal disturbances, in particular hypogonadism. Diseases commonly associated with type 2 diabetes, such as arterial hypertension, atherosclerosis, hyperlipidaemia and metabolic syndrome, also play an important role in the development of hypogonadism. These further contribute to the development of hypogonadism through their effects on insulin, leptin and inflammatory cytokines. Hypogonadism causes a decrease in testosterone levels, which has direct consequences not only on libido but also on the psychological state of the male patient—leading to depression, anxiety and poorer sleep quality. Reduced testosterone further contributes to the development of ED, alongside functional and structural changes in the vasculature, nerves and smooth muscle tissue, as well as decreased levels of nitric oxide (NO), which is essential for penile muscle relaxation and an adequate haemodynamic response. These interconnected disorders contribute to a complex clinical presentation of diabetes‐related ED. Consequently, effective management requires a holistic approach that considers the patient's hormonal, neurovascular and psychological health [26, 27, 28, 29, 30]. AGE, advanced glycation end‐products; NO, Nitric oxide.

The pathophysiology of diabetic ED may be mediated by endothelial dysfunction, vasculopathy, neuropathy, hormonal imbalances, psychological factors and histological changes in penile tissue. The interplay of these factors is summarised in Figure 1.

#### 2.4.1 Impaired Endothelial Function

Cavernosal endothelial dysfunction is a hallmark of diabetic ED, as the intact endothelium plays a critical role in regulating penile vascular tone. Chronic hyperglycaemia increases the formation of advanced glycation end‐products (AGEs), leading to reduced NO bioavailability and increased oxidative stress within endothelial cells [31, 32]. Furthermore, endothelial NO synthase (eNOS) expression and activity are often downregulated in patients with DM, hindering penile vasodilation and impairing the erectile response [33].

Endothelium‐derived hyperpolarising factor (EDHF) signalling dysfunction may also contribute to impaired vasodilation in diabetic ED. Although NO is the primary mediator of endothelium‐dependent smooth muscle relaxation in both the corpus cavernosum and penile arteries, EDHF may play a particularly significant role in the penile arteries. This potential role of EDHF in vasodilation may explain the reduced responsiveness to PDE5 inhibitors observed in some patients with DM. Supporting this hypothesis, a study by Angulo et al. on 33 human penile tissue samples obtained via biopsy demonstrated that DM impairs the vasodilatory response to EDH [32, 34].

#### 2.4.2 Atherosclerosis

Studies suggest a significant role of atherosclerosis in the pathophysiology of ED, with ED serving as a predictor of future cardiovascular events [35]. ED can be an early manifestation of coronary heart disease or peripheral arterial disease, representing not just a quality‐of‐life issue but also a warning sign of underlying CVD [36]. The presence of ED is associated with an increased risk of future cardiovascular events (e.g., myocardial infarction, cerebrovascular events) and overall mortality [15].

Because atherosclerosis is a systemic process, it can impair the hypogastric‐cavernous‐helicine arterial tree, reducing perfusion pressure and blood flow to the sinusoidal spaces [17]. Furthermore, atherosclerosis lowers penile oxygen tension [37], which may negatively impact penile function, as higher oxygen tension is associated with increased PGE_2_ levels and suppression of collagen synthesis [38].

In patients with DM, hyperglycaemia and other associated factors accelerate atherosclerosis [39, 40]. A study by Gazzaruso et al. [41] investigated ED as a predictor of future cardiovascular events and mortality in patients with DM and silent coronary artery disease. The study found that ED was more prevalent in patients who experienced major adverse cardiac events during the follow‐up period (61.2% vs. 36.4%). Another study in men with T2DM demonstrated that those with ED had increased carotid intima‐media thickness and a higher prevalence of lower‐limb plaques compared to those without ED [42].

#### 2.4.3 Neuropathy

In DM, chronic hyperglycaemia leads to the formation of AGEs and increased oxidative stress. Furthermore, the pathogenetic mechanisms underlying diabetic neuropathy involve increased flux through the polyol pathway, dysregulation of vasoactive substances, non‐enzymatic glycation and altered neurotrophism [43, 44]. These processes contribute to demyelination and axonal degeneration in both somatic peripheral nerves and the autonomic nervous system.

Autonomic neuropathy, a common complication of DM, contributes to ED by disrupting parasympathetic function, which is essential for smooth muscle relaxation and erection [45, 46, 47]. Additionally, peripheral neuropathy impairs sensory input from the penis, compromising activation of the reflexogenic erectile centre. This input is critical for erection in response to genital stimulation, provided the S2 to S4 spinal centre remains intact [48, 49, 50, 51, 52]. Furthermore, efferent pudendal nerve signals are vital for muscle contractions that enhance erectile rigidity [53, 54, 55].

A study by Hicks et al. [56] involving 1213 patients demonstrated an association between decreased lower‐extremity sensation and ED. Another study of 70 patients with T1DM and 34 age‐matched controls showed an association between small‐fibre neuropathy and ED [57]. Similarly, the Dogo study on Japanese patients found an association between diabetic neuropathy and ED, but not between ED and nephropathy or retinopathy [58].

#### 2.4.4 Structural Changes of the Penile Tissue

Erectile function depends on the compliance of penile sinusoids, which is conferred by elastic fibres [17]. In DM, however, the amount of elastic fibres is reduced, accompanied by increased collagen deposition [59, 60]. These changes, along with alterations in smooth muscle, affect sinusoidal histology and function [61].

In DM, oxidative stress and inflammation can impair matrix metalloproteinase (MMP) activity, resulting in ECM imbalance and fibrosis, which contribute to fibroproliferative dysfunction. Furthermore, DM‐related alterations in MMP activity may impair smooth muscle cell function, reducing structural elasticity and thus decreasing blood flow to the corpora cavernosa [62, 63]. A study by Muniz et al. [64] demonstrated reduced net matrix metalloproteinase‐9 (MMP‐9) activity in patients with diabetic ED, but no significant changes in circulating levels of MMP‐9 or MMP‐2.

Degenerative changes in the tunica albuginea and other penile tissues may lead to insufficient compression of the emissary and subtunical veins, impairing the veno‐occlusive mechanism [17, 62]. Genome‐wide association studies (GWAS) and gene expression analyses have demonstrated that DM may dysregulate fibroproliferative processes, similar to those observed in Peyronie's disease and Dupuytren's contracture, both of which are recognised risk factors for ED [65, 66]. Furthermore, chronic hyperglycaemia can lead to tissue injury and subsequent fibrosis. DM itself is also a risk factor for Peyronie's disease and Dupuytren's contracture [62, 66, 67].

#### 2.4.5 Psychological Factors

Numerous biological mechanisms may link psychological factors to ED. For example, Hong et al. [68] investigated the mechanisms underlying ED in a rat model of depression and found that ED was associated with dopamine system dysfunction. Other studies have focused on structural and functional neuroanatomy in patients with psychogenic ED [69, 70, 71, 72, 73, 74, 75, 76]. However, the precise role of the central nervous system in ED remains largely unknown. From a psychological perspective, anxiety during sexual activity can create cognitive distraction, thereby affecting arousal and erection [77]. Furthermore, a disproportionate anxiety response can increase sympathetic tone, impairing the physiological mechanisms underlying erection [78, 79]. Because ED itself can worsen negative self‐image and cause considerable psychological stress, a vicious cycle can develop [80].

The significance of psychological factors in diabetic ED remains incompletely understood. While researchers generally agree that organic factors are the primary determinants of ED in patients with DM [10, 81], some studies suggest a role for psychological factors as well. For instance, the presence of nocturnal and morning erections in some men with diabetic ED suggests that psychological factors may be significant in this pathology [47, 82, 83]. A multicentre study by De Berardis et al. [84] involving 1460 patients with T2DM found that severe ED correlated with T2DM severity, whereas mild‐to‐moderate ED was associated with the severity of depressive symptoms rather than the clinical severity of T2DM.

A meta‐analysis by Chireh et al. [85] revealed that the risk of depression is 1.33 times higher in people with DM. Multiple aspects of depression may contribute to ED in these patients. A separate meta‐analysis reported that individuals with both DM and comorbid depression were significantly more likely to experience ED compared to those without depression (74.2% vs. 37.4%, respectively) [86]. A cross‐sectional study by Raghuraman et al. [87] of 70 younger men (aged 20–40 years) with T2DM found that hypoactive sexual desire disorder was present in 44.28% of participants, followed closely by ED (42.85%) and ejaculatory disorders (18.57%). Another study by Malavige et al. [82] involving 253 patients with T2DM demonstrated a strong association between ED, premature ejaculation and reduced libido.

Furthermore, antidepressants, particularly SSRIs, serotonin‐norepinephrine reuptake inhibitors (SNRIs) and tricyclic antidepressants (TCAs), can impair sexual function. Consequently, patients who develop ED while taking these medications may exhibit reduced adherence to treatment [88, 89, 90].

Emerging research suggests a possible link between circadian rhythm disruption and ED. Shift workers, who often experience circadian disruption, are more likely to report fatigue and exhibit impaired metabolic homeostasis. Poor sleep quality, a common consequence of shift work, has been shown to predict hypogonadal symptoms and sexual dysfunction in male shift workers [91, 92]. Furthermore, circadian rhythm disruption itself increases the risk of both DM [93, 94, 95] and ED in the general population [96, 97, 98], suggesting a shared risk factor for these conditions.

#### 2.4.6 Hormonal Factors

Hypogonadism, which is frequently associated with DM, is another important factor in the pathogenesis of diabetic ED. Chronic hyperglycaemia impairs the hypothalamic–pituitary–testicular (HPT) axis, leading to functional hypogonadism (often termed late‐onset hypogonadism). While the precise mechanisms underlying this dysfunction remain unclear, HPT‐axis suppression may be caused by the presence of anti‐pituitary antibodies and an imbalance of pro‐inflammatory factors, potentially leading to hypothalamic inflammation [99, 100]. Additionally, sex hormone‐binding globulin (SHBG) levels are often reduced in DM, while aromatase activity is increased. These factors, along with dysregulation of leptin, insulin and oestradiol signalling, contribute to reduced androgen action in patients with DM [100, 101]. Conversely, associated metabolic risk factors may also contribute to hypogonadism, and lower testosterone levels are themselves a risk factor for the development of T2DM [102].

As a result of HPT‐axis suppression, these patients experience androgen deficiency with non‐elevated luteinising hormone (LH) levels. Consequently, this androgen deficiency may contribute to ED development through various pathological mechanisms [100].

Testosterone enhances vascular tone and NOS activity and supports nerve function while promoting pro‐erectile neurotransmitter release in the CNS [47, 103, 104, 105]. Androgen deficiency may therefore lead to penile tissue atrophy, reduced smooth muscle content in the trabeculae, increased ECM deposition, subtunical adipocyte accumulation, dorsal nerve structural changes and altered endothelial morphology [106]. Furthermore, testosterone deficiency contributes to reduced muscle strength and mass, along with increased central adiposity, which may negatively affect psychological well‐being and aggravate several obesity‐related pathological pathways [107]. Decreased serum testosterone levels are common in patients with T2DM, with a high prevalence of hypogonadism (approximately 30%–40%) reported [108, 109, 110, 111]. However, a recent meta‐analysis indicated that T2DM alone has a modest effect on the relative reduction in total testosterone, with increased fat mass being a more significant contributing factor [101, 112, 113]. In contrast, subnormal testosterone levels are relatively uncommon in men with T1DM without obesity [100]. Nevertheless, poor glycaemic control is associated with greater HPT‐axis suppression [101, 112, 113]. A study by Raghuraman et al. [87] found a significant correlation between hypogonadism and hypoactive sexual desire disorder in young men with T2DM. This study also found an association between neuropathy and ED in these patients. Hypogonadism also contributes to various other conditions that can indirectly affect erectile function. It can lead to decreased physical energy, reduced motivation, irritability, sleep disturbances and depressed mood in men [114].

Interestingly, ED could serve as an early clinical indicator of underlying endocrinopathies. Mazzilli et al. [115] evaluated the prevalence of previously undiagnosed endocrine and glycaemic disorders in 1332 men with ED and examined the correlation between these disorders and ED severity. Among patients without known conditions, 30% had new endocrine or glycaemic abnormalities, including hypogonadism (59%), diabetes/prediabetes (17%), thyroid dysfunction (12%) and hyperprolactinaemia (12%). Newly diagnosed diabetes was significantly associated with more severe ED, particularly difficulty achieving erection and lack of spontaneous erections. Moreover, Chen et al. [116] found a high prevalence of subclinical hypothyroidism (SCH) in men with ED, with thyroid dysfunction and elevated prolactin linked to lower erectile scores. Although SCH did not worsen ED severity, its presence in ED patients suggests that thyroid screening is important. Further research is needed to determine whether routine endocrine screening in ED patients is warranted, as ED might be a sensitive but non‐specific marker of various endocrinopathies.

### 2.5 Genetic Background and Pharmacogenetics of Erectile Dysfunction in Patients With Diabetes Mellitus

While the pathophysiology of diabetic ED is multifactorial, involving vascular, neural, hormonal and structural changes, a growing body of evidence suggests a significant role for genetic factors in both disease susceptibility and individual responses to treatment. Understanding the genetic associations of diabetic ED is crucial for identifying individuals at increased risk, elucidating underlying disease mechanisms and potentially tailoring therapeutic approaches. This section will explore the genetic background of diabetic ED, focusing on key candidate genes and their associated polymorphisms that have been linked to ED risk, severity and treatment outcomes. Table 1 summarises these genetic associations, their pathogenetic significance and pharmacogenetic implications.

**TABLE 1:** Summary of key genes and polymorphisms in diabetic erectile dysfunction, their pathophysiological mechanisms and associations and pharmacogenetic implications.

Gene and key references	Polymorphisms	Mechanistic basis	Pathogenetic significance	Clinical and pharmacogenetic implications
***NOS3 (eNOS)** (Endothelial Nitric Oxide Synthase)* [1, 2, 3, 4, 5, 6, 7, 8, 9]	G894T (Glu298Asp) T786C 27‐bp VNTR in intron 4	Altered eNOS activity reducing NO/cGMP pathway efficiency	Influences NO production, critical for penile smooth muscle relaxation. Certain variants (e.g., G894T, T786C) linked with altered NO synthesis, ED risk/severity and possible earlier ED onset. Can affect PDE5I efficacy because eNOS‐derived NO is essential for cGMP‐mediated vasodilation	Potential biomarker for PDE5I response (e.g., carriers of G894T or T786C might differ in treatment outcomes). Could guide personalised PDE5I therapy or adjunct strategies targeting NO pathways in diabetic ED management. Indicates a complex, ethnicity‐dependent risk profile
***ARG1/2*** *(Arginase 1/2)* [10, 11, 12, 13, 14, 15, 16, 17, 18]	ARG1/ARG2 variants (exact SNPs often population‐specific) affecting gene expression and enzyme activity	Increased arginase activity competes with NOS for L‐arginine	Compete with NOS for L‐arginine, reducing NO production. Upregulated ARG2 in diabetes linked to endothelial dysfunction and decreased penile vasodilation. ARG1 variants also associated with severity of diabetic vascular complications (e.g., retinopathy). Involvement of ERK‐arginase, ROCK2 and p38 MAPK pathways further impairs cavernosal relaxation	Arginase inhibitors or related pathway modulators (e.g., ERK, ROCK2/p38 MAPK) may be more beneficial in patients with high arginase activity/genetic risk. Pharmacogenetic profiling might identify those most likely to respond to ARG inhibition or combined PDE5I + arginase blockade
***ACE** (Angiotensin‐Converting Enzyme)* [19, 20, 21, 22, 23, 24]	I/D polymorphism (intron 16), characterised by the I (insertion) or D (deletion) allele	Modulation of RAAS pathway, with altered angiotensin II levels influencing vascular tone	Influences penile vascular tone and smooth muscle function. The D allele sometimes linked to greater vascular risk (e.g., micro‐/macrovascular disease) and earlier ED onset in certain populations. I allele associated with ED complaints. Inconsistent ED associations across studies, possibly due to ethnic/genetic heterogeneity	Determining the ACE I/D genotype may help tailor RAAS‐modulating therapies (ACE inhibitors/ARBs) in patients with ED and cardiovascular comorbidities. Conflicting results suggest population‐specific genetic relevance
***AR CAG Repeat** (Androgen Receptor)* [25, 26, 27, 28, 29, 30, 31]	CAG repeat length polymorphism in exon 1	Modulation of androgen receptor transcriptional activity affecting androgen sensitivity	Longer CAG repeats→decreased androgen sensitivity; may worsen ED by impairing testosterone's actions. Combined with metabolic dysfunction, longer repeats can exacerbate cardiometabolic risks and negatively impact erection physiology. Shorter repeats are linked to lower risk of hypogonadism and insulin resistance	Testosterone therapy optimisation (e.g., dose adjustments) for individuals with longer AR CAG repeats who have suboptimal androgen signalling. Pharmacogenetic profiling might help predict which patients require higher testosterone doses or adjunct PDE5I/endothelial therapies to achieve erectile function
***VEGF** (Vascular Endothelial Growth Factor)* [32, 33, 34, 35, 36, 37]	(−2578C>A) (−1154G>A) Other promoter haplotypes (e.g., “AA”)	Altered VEGF expression affecting angiogenesis and vascular integrity	Regulates endothelial cell proliferation and penile vascular remodelling Influences intracavernosal pressure and tissue remodelling; modulation of the eNOS pathway is critical for vascular restoration in ED The −2578A allele is associated with increased ED prevalence and severity, notably in metabolic syndrome Promoter haplotypes (−1154AA, −2578AA) linked with poorer PDE5I responsiveness	VEGF genotyping might identify patients at risk of poor response to PDE5I therapy (e.g., carriers of −2578AA, −1154AA). Could inform combination therapies (e.g., angiogenic agents, endothelial modulators) to augment PDE5Is in patients with unfavourable VEGF polymorphisms

Abbreviations: ACE, angiotensin‐converting enzyme; AR, androgen receptor; ARBs, angiotensin II receptor blockers; ARG, arginase; cGMP, cyclic guanosine monophosphate; DM, diabetes mellitus; ED, erectile dysfunction; eNOS/NOS3, endothelial nitric oxide synthase; ERK, extracellular signal‐regulated kinase; I/D, insertion/deletion; MAPK, mitogen‐activated protein kinase; NO, nitric oxide; PDE5, phosphodiesterase type 5; PDE5Is, phosphodiesterase type 5 inhibitors; RAAS, renin‐angiotensin‐aldosterone system; ROCK, Rho‐kinase; SNP, single nucleotide polymorphism; VEGF, vascular endothelial growth factor; VNTR, variable number tandem repeat.

#### 2.5.1 *NOS3* (eNOS) Gene

The *NOS3* gene, encoding endothelial nitric oxide synthase (eNOS), is a key candidate gene in the relaxation pathway of penile smooth muscle and is located in the 7q35 to 7q36 region of chromosome 7. Three isoforms of NOS are known: neuronal NOS (nNOS or NOS1), inducible NOS (iNOS or NOS2) and endothelial NOS (eNOS or NOS3). Rapid activation of nNOS is thought to initiate erection, while phosphorylation and activation of eNOS maintain it [117]. *NOS3* polymorphisms occur with varying frequencies among different ethnic groups [118].

Numerous studies have shown that functional polymorphisms of the *NOS3* gene are associated with impaired erectile function, reduced NO synthesis, and decreased cGMP levels, all contributing to an increased risk of ED [119]. One of the first studies to address *NOS3* polymorphisms in the context of PDE5I treatment was by Eisenhardt et al. This study included 113 men with ED and 108 healthy controls. The researchers analysed the response to sildenafil treatment in the ED group, assessing improvements in erectile function. *ACE* and *NOS3* genotypes were determined in both groups. The distribution of *ACE* and *NOS3* polymorphisms was similar between the groups. Analysis of sildenafil response revealed a beneficial effect on erectile function in 15 of 20 patients homozygous for the *ACE* II genotype and in 30 of 52 patients homozygous for the *NOS3* 894T allele. Polymorphisms in the *PDE5A* gene, which encodes PDE5, have been associated with a reduced response to NO and altered cGMP levels, potentially influencing the binding capacity of PDE5Is. Additionally, *VEGF*, a cytokine involved in angiogenesis, is closely related to the NO‐cGMP signalling pathway targeted by sildenafil [31].

Multiple meta‐analyses have investigated the link between three common NOS3 polymorphisms and the risk of ED. The specific variants studied are G894T (also known as Glu298Asp), a 27‐bp VNTR in intron 4, and T786C. However, the findings from these analyses have been inconsistent [120, 121, 122, 123, 124]. The G894T and T786C polymorphisms, in particular, have been frequently implicated. Different meta‐analyses report a significant association between G894T and increased ED risk, particularly under allele and dominant models [120, 122, 124], and another reported it with a decreased risk [123]. The meta‐analysis specifically focusing on the G894T polymorphism, found a significantly decreased association with ED risk in both Caucasian and Asian subgroups [123]. The T786C polymorphism has also been significantly associated with increased ED risk in several analyses [120, 121, 124]. The intron 4 VNTR polymorphism's association with ED has been less consistent, with some studies showing an association, particularly in Caucasian populations [121], and others finding no significant association [122]. This variability in risk association underscores the pharmacogenetic complexity of NOS3 polymorphisms, which may influence both baseline ED susceptibility and therapeutic responses to NO‐enhancing therapies.

Beyond overall risk, some studies have explored the impact of NOS3 polymorphisms on ED characteristics. In a Han Chinese population, the G894T polymorphism was associated with an earlier age of ED onset, and the T786C polymorphism was correlated with greater ED severity, as measured by the IIEF‐5 score [125]. These findings suggest that NOS3 variations may not only influence susceptibility to ED but also affect its clinical presentation. The conflicting results across these meta‐analyses and variable outcomes for different models emphasise the need for larger, well‐designed studies, possibly accounting for factors like ethnicity and gene–environment interactions. Given the central role of eNOS in NO production and the reliance of PDE5Is on sufficient NO levels, these *NOS3* polymorphisms could potentially serve as pharmacogenomic markers to predict individual responses to PDE5I therapy, although further research is needed to confirm this.

#### 2.5.2 *ARG1/ARG2* Gene

Arginase 1 (ARG1) and arginase 2 (ARG2) are enzymes that utilise L‐arginine as a substrate. Unlike nitric oxide synthase (NOS), which uses L‐arginine to produce NO and L‐citrulline, arginases stimulate the synthesis of L‐ornithine and urea, thereby competing with NOS isoforms (nNOS/NOS1 and eNOS/NOS3) for L‐arginine. Both ARG1 and ARG2 are expressed in the corpus cavernosum and localised to the endothelium, similar to eNOS. This competition for L‐arginine means that increased ARG activity results in reduced NO synthesis due to substrate depletion. Lacchini et al. [126] concluded that plasma ARG2 levels may be a risk factor for ED, while *ARG1* genetic variations may affect ED severity.

Increased arginase activity, specifically the ARG2 isoform, has been directly implicated in the pathogenesis of diabetic ED. Studies have demonstrated elevated ARG2 protein levels, gene expression and enzyme activity in cavernosal tissue from diabetic men with ED compared to nondiabetic controls [127]. This upregulation of *ARG2* in DM is believed to contribute to endothelial dysfunction by reducing L‐arginine availability for eNOS, thus impairing NO‐mediated vasodilation, a critical process for penile erection. Pharmacogenetic insights into *ARG2* polymorphisms could help identify patients who may benefit most from arginase inhibitors, which are emerging as a novel therapeutic strategy for diabetic ED [128].

The link between arginase and ED extends beyond direct substrate competition. Studies in diabetic mouse models have shown that inhibiting extracellular signal‐regulated kinase (ERK) not only decreases arginase activity but also improves cavernosal relaxation [129]. Furthermore, genetic deletion of *ARG2* has been shown to enhance corpora cavernosa relaxation in diabetic mice, further supporting *ARG2*'s role in diabetic ED [130]. These findings suggest that targeting the ERK‐arginase pathway could be a promising pharmacogenetic approach, particularly in patients with specific *ARG2* polymorphisms associated with elevated arginase activity. The RhoA/Rho‐kinase (ROCK) pathway, specifically ROCK2, and p38 MAPK have also been identified as upstream mediators of DM‐induced arginase elevation and impaired cavernosal relaxation [131].

*ARG1* polymorphisms have also been associated with an increased risk of diabetic retinopathy in T2DM patients [132], and *ARG1* genetic variations have been linked to vascular complications in T2DM, suggesting a broader role for arginase in diabetic vascular disease [133]. In Akita type 1 diabetic mice, elevated arginase activity and expression (both ARG1 and ARG2 in corpora cavernosa) were observed alongside impaired endothelial and nitrergic function, further supporting a mechanistic link [134]. Targeting arginase, therefore, presents a potential therapeutic strategy for ED and potentially other vascular complications of DM [133, 134]. Pharmacogenetic profiling of *ARG1* and *ARG2* polymorphisms could help stratify patients for targeted therapies, such as arginase inhibitors or ROCK2/p38 MAPK pathway modulators, optimising treatment outcomes in diabetic ED.

#### 2.5.3 *ACE* Gene

ACE is a central component of the renin–angiotensin–aldosterone system (RAAS), primarily involved in regulating blood pressure and fluid balance, rather than directly controlling mineralocorticoid synthesis. The *ACE* gene contains an insertion/deletion (I/D) polymorphism, characterised by the presence (insertion, I allele) or absence (deletion, D allele) of a 287‐bp DNA sequence in intron 16. Penile smooth muscle tone and contractility are partly regulated by vasoactive substances, including angiotensin II, which is a product of the RAAS pathway [135]. *ACE I/D* polymorphism may affect the response to RAAS‐modulating therapies, such as ACE inhibitors or angiotensin receptor blockers (ARBs), potentially influencing ED management in patients with comorbid hypertension or CVD. Therefore, evaluating the *ACE I/D* genotype in men with ED could refine individualised therapeutic approaches.

Several studies have attempted to elucidate the relationship between the *ACE I/D* polymorphism and ED risk, yielding mixed results. For instance, in a study of Russian men with metabolic syndrome, the DD genotype was significantly more prevalent in those with ED compared to those without ED. Furthermore, in this cohort, men with the DD genotype exhibited an earlier age of ED onset [136]. A Brazilian study also found a significant association between the I allele (specifically, the ID and II genotypes) and ED complaints in men aged 40–55 years, even after adjusting for potential confounders, including genetic ancestry [137].

However, other studies, such as one conducted on a German population, did not find a significant association between the *ACE* I/D polymorphism and ED risk, age of onset, or response to prostaglandin injection [138]. Zhang et al. [139] conducted a meta‐analysis of six case–control studies, including 1039 ED cases and 927 healthy controls, and found no significant association between the *ACE* I/D polymorphism and ED risk. These conflicting findings may be attributable to differences in populations studied (e.g., ethnicity, presence of comorbidities like metabolic syndrome), sample sizes, or methodological variations. Nevertheless, the findings underscore the importance of considering population‐specific genetic backgrounds and environmental factors when interpreting the pharmacogenetic relevance of *ACE* polymorphisms in ED. Meanwhile, it is also crucial to note that the D allele, although sometimes associated with ED, is also linked to increased risk of micro‐ and macrovascular diseases in general [136]. The precise mechanism by which the *ACE* I/D polymorphism might influence ED risk remains to be fully elucidated but likely involves alterations in angiotensin II levels and subsequent effects on vascular tone and smooth muscle function.

#### 2.5.4 *AR* CAG Repeats

Testosterone plays an important role in male sexual function, but its effects are modulated by the CAG repeat region within the androgen receptor (*AR*) gene [140]. The *AR* gene contains a polymorphic CAG repeat sequence in exon 1 and the length of this repeat tract has been shown to modulate androgen sensitivity. A longer CAG repeat generally correlates with reduced transcriptional activity of the AR, meaning that a higher number of repeats can effectively diminish the cellular response to testosterone.

Several investigations indicate that variations in the CAG repeat length within the *AR* gene modulate androgen responsiveness and can affect the development or severity of ED [141]. Some evidence suggests that shorter CAG repeats are associated with a reduced long‐term risk of developing low testosterone. In contrast, longer expansions may exacerbate insulin resistance and cardiometabolic complications, particularly when this occurs in the context of poor metabolic control [142, 143]. These findings align with mechanistic insights that AR function influences metabolic pathways and endothelial physiology, both of which are central to erectile function. Liu et al. [144] concluded that both serum testosterone levels and *AR* CAG repeat length can influence erectile function. In individuals with normal total testosterone levels, those with longer *AR* CAG repeats have a higher risk of ED. This suggests that *AR* CAG repeat length may influence the efficacy of testosterone replacement therapy and could serve as a pharmacogenetic marker to identify patients who may require higher doses of testosterone or adjunct therapies to achieve optimal erectile function.

Nevertheless, variability exists, as certain populations show minimal correlation between CAG length and ED indices, pointing to the influence of factors such as comorbid hypogonadism or systemic disorders [145]. Moreover, the local expression of downstream factors involved in vascular and smooth muscle function can further modulate AR‐driven pathways. This suggests that the net impact of the *AR* CAG repeats on ED is not determined by a single factor but rather by a complex interplay among hormonal status, receptor sensitivity and cardiovascular risk profiles [146]. Collectively, the evidence suggests that the *AR* genotype may help predict treatment outcomes. Pharmacogenetic profiling of *AR* CAG repeat length, combined with cardiovascular risk assessment, could aid in stratifying patients for targeted therapies such as PDE5Is or endothelial modulators, particularly in cases of hypogonadism‐related ED [145, 146].

#### 2.5.5 *VEGF* Gene

VEGF plays a crucial role in penile vasculature and erectile function by regulating physiological pathways involved in penile vasomotor tone. Multiple lines of evidence indicate that VEGF not only stimulates endothelial cell proliferation but also modulates pathways essential for restoring cavernous smooth muscle integrity, particularly in DM‐related ED [147, 148]. In vivo administration of VEGF, either directly or via gene delivery, enhances intracavernous pressure and promotes favourable tissue remodelling. These effects appear to involve several molecular mechanisms, including the regulation of insulin‐like growth factor signalling, changes in sex hormone receptor expression and upregulation of the eNOS pathway. This evidence suggests that VEGF‐based therapeutic strategies could counteract the vascular and endothelial impairments characteristic of diabetic ED [148, 149].

Lee et al. [150] investigated three *VEGF* polymorphisms and their association with ED. They found that the prevalence and severity of ED were significantly increased with an increasing number of the −2578A allele. Recent findings also highlight the importance of VEGF polymorphisms in predicting both disease risk and treatment response. A notable example is the 2578C>A variant, where the A allele has been linked to a higher prevalence of ED in men with metabolic syndrome [151]. In addition, patients harbouring specific VEGF promoter haplotypes, including −1154AA and −2578AA, may exhibit poorer responsiveness to PDE5Is [152]. These observations underscore the multifactorial role of VEGF in ED pathogenesis, indicating that not only are VEGF‐mediated pathways critical for vascular restoration, but genetic variants can further modulate therapeutic efficacy and disease progression. The association between specific *VEGF* polymorphisms and altered responses to PDE5Is, such as sildenafil, suggests that *VEGF* genotyping could potentially be used to personalise ED treatment by identifying patients who may be less likely to respond to standard PDE5I therapy.

#### 2.5.6 FIBROSIS‐Related Genes

The study by Deng et al. [153], conducted on rat models, investigated the genetic profile of fibrosis and explored potential mechanisms underlying diabetic ED. A total of 45 differentially expressed fibrosis‐related genes involved in smooth muscle cell proliferation, vasoconstriction and collagen‐associated processes were identified. The analysis revealed that a gene signature consisting of TIMP1, BMP7 and POSTN is closely associated with signalling pathways and extracellular matrix–receptor interactions. A summary of key genes and polymorphisms in diabetic ED, their pathophysiological mechanisms and associations, and pharmacogenetic implications is shown in Table 1.

### 2.6 Clinical Presentation and Assessment of ED in Patients With Diabetes Mellitus

The initial evaluation of a patient with ED should include a comprehensive medical history, with a particular focus on sexual history. Involving the patient's partner in this process, when feasible, is also beneficial [154]. The history should include a detailed description of the rigidity and duration of both sexually stimulated and morning erections, as well as any difficulties with sexual desire, arousal, ejaculation, or orgasm [155]. Validated psychometric questionnaires, such as the International Index of Erectile Function (IIEF) or its abbreviated version, the Sexual Health Inventory for Men (SHIM), are valuable tools for assessing the various domains of sexual function (e.g., sexual desire, orgasmic function, intercourse satisfaction and overall satisfaction) and for evaluating the potential impact of treatment [156, 157]. Patients should be screened for symptoms of hypogonadism, including decreased libido, reduced energy levels and fatigue.

All patients require a focused physical examination, including genitourinary, endocrine, vascular and neurological systems. Physical examination may reveal previously undiagnosed conditions, such as Peyronie's disease, premalignant or malignant genital lesions, prostatic enlargement or irregularity/nodularity, or signs and symptoms suggestive of hypogonadism. If not assessed within the previous 12 months, patients should undergo fasting blood glucose or HbA1c testing, along with a lipid profile. Hormonal testing should include an early‐morning, fasting total testosterone measurement. Bioavailable or calculated free testosterone (fT) levels may be needed to corroborate total testosterone, particularly if total testosterone is borderline or if SHBG abnormalities are suspected. Additional laboratory tests, such as prostate‐specific antigen (PSA), prolactin and luteinising hormone, may be considered in selected patients based on specific signs, symptoms, or risk factors [158].

Additional diagnostic testing may include nocturnal penile tumescence and rigidity (NPTR) monitoring, which uses specialised devices to measure the number of erectile episodes, penile circumference changes (tumescence), maximal penile rigidity and duration of nocturnal erections [159]. Nocturnal penile tumescence and rigidity (NPTR) monitoring can help differentiate between organic and psychogenic ED; patients with psychogenic ED typically have normal NPTR results [160]. The intracavernosal injection (ICI) test provides limited information about vascular status. A positive ICI test is defined as a rigid erectile response (inability to bend the penis) that develops within 10 min of injection and lasts for at least 30 min [154]. However, the ICI test alone is often inconclusive. Penile duplex Doppler ultrasound (US) is a second‐line diagnostic test that specifically assesses the haemodynamic pathophysiology of ED. Therefore, it is typically used when a vasculogenic aetiology is suspected [161].

### 2.7 Treatment Options

#### 2.7.1 Non‐Pharmacological Treatments and Lifestyle Interventions

Patient education is often the initial approach to addressing sexual concerns and involves explaining the psychological and physiological processes involved in sexual response in an accessible manner. The above‐mentioned method has been shown to improve sexual satisfaction in men with ED [162]. ED may be associated with modifiable or reversible risk factors, including lifestyle and medication‐related factors [163]. These risk factors can be addressed either before or concurrently with specific ED therapies. The pathology has been associated with underlying and coexisting conditions (e.g., endocrine and metabolic disorders such as DM and CVDs such as hypertension), which should be optimally managed as a first step in any ED treatment plan [164]. Studies have shown that lifestyle modifications, including increased physical activity, weight loss and management of cardiovascular risk factors, can improve sexual function in men with ED [163].

Psychosexual counselling or sex therapy can be beneficial for patients experiencing relationship discord, particularly if the conflict is related to the patient's ED. These therapies typically involve 5–20 sessions. Therapy is unlikely to be effective with premature discontinuation (e.g., after only one or two sessions). Studies have shown that for patients with stress‐related ED, partner involvement in therapy is associated with a 50%–70% resolution rate [165].

A retrospective study conducted by Zamponi et al. [166] found that the prevalence of non‐response to PDE5 inhibitors was higher among patients treated with older antihyperglycemic agents compared to those receiving newer therapies. The authors concluded that improved glycometabolic control, along with the use of newer antihyperglycemic drugs, appears to have a beneficial effect on ED.

#### 2.7.2 Current Pharmacological Treatments

The European Association of Urology (EAU) Guidelines Panel has developed a comprehensive therapeutic and decision‐making algorithm for managing ED. The majority of patients with ED are not treated with cause‐specific therapeutic options. This necessitates a tailored treatment approach that considers invasiveness, efficacy, safety, cost and patient preference; therefore, physician‐patient communication is essential throughout the management of ED [167]. A prospective observational study by Defeudis et al. [168] in T2DM patients with ED revealed that health literacy, treatment adherence, unrealistic optimism and glycaemic control are interrelated. Patients with lower health literacy had higher BMI and IIEF‐5 scores, while those on insulin showed better health literacy. Higher adherence was observed in patients using SGLT2 inhibitors, and GLP‐1 receptor agonist use was associated with better erectile function.

##### 2.7.2.1 Phosphodiesterase Type 5 Inhibitors (PDE5I)

Phosphodiesterase type 5 (PDE5) is a multidomain protein comprising a regulatory and a catalytic domain. The regulatory domain, located at the N‐terminus, consists of two tandem GAF domains (GAFa and GAFb) that control catalytic activity and protein dimerisation. While multiple genes can encode specific PDE family members, *PDE5A* is the only gene that encodes the PDE5 protein, which is expressed as three isoforms: PDE5A1, PDE5A2 and PDE5A3. These isoforms share an identical catalytic domain but differ in the length of their N‐terminal regulatory domain. PDE5A1 and PDE5A2 are widely expressed, whereas PDE5A3 is primarily expressed in vascular smooth muscle cells, providing an additional layer of regulation [169]. Competitive PDE5Is bind exclusively to the catalytic domain, preventing cyclic GMP (cGMP) binding and its subsequent hydrolysis. This inhibition leads to cGMP accumulation, resulting in various therapeutic benefits. In addition to cGMP‐mediated allosteric activation, PDE5 function is regulated at the genetic level by isoform expression and post‐translationally by modifications such as phosphorylation (which activates PDE5) and *S*‐nitrosylation (which targets it for degradation via the ubiquitin‐proteasome pathway) [170].

PDE5 inhibitors competitively and reversibly inhibit PDE5 in the corpora cavernosa, the enzyme responsible for cGMP hydrolysis. This inhibition potentiates the effects of NO‐mediated cGMP production, leading to increased cGMP levels and prolonged penile erection [169].

Four potent, selective PDE5Is (sildenafil, tadalafil, vardenafil and avanafil) have been approved by the European Medicines Agency (EMA) for the treatment of ED [171]. The efficacy of all four PDE5Is has been established across almost all subgroups of patients with ED. These medications improve erectile function in the majority of patients by enhancing blood flow to the corpora cavernosa [171]. While all are effective, they differ in their selectivity, potency, indications, onset and duration of action, cost, administration, precautions and adverse effect profiles. Vardenafil has been reported to have the highest relative affinity for PDE5, followed by tadalafil and then sildenafil. This greater in vitro potency of vardenafil has been attributed to its heterocyclic double‐ring structure. The detailed characteristics of these four PDE5 inhibitors, including their pharmacological properties, efficacy and administration, are summarised in Table 2.

**TABLE 2:** Comparative overview of PDE5 inhibitors (PDE5Is) for erectile dysfunction.

PDE5 inhibitor (*brand name and year of approval*)	Chemical class/structure^a^	Molecular interactions/mechanisms	Dosing	Pharmacokinetics	Efficacy/Dose–response outcomes
Sildenafil *1998*	Sulfonamide derivative 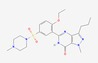	Binds to PDE5's catalytic domain via the Q‐pocket, H‐pocket and L region; does not interact directly with the M subsite or its coordinating Zn^2+^/Mg^2+^; Moderate selectivity for PDE5 over PDE6; Heterocyclic ring mimics cGMP purine; The pyrazole N2 atom forms a hydrogen bond with a water molecule that in turn bonds with Tyr612 (Q‐pocket) and a Zn^2+^‐coordinated water	Starting Dose: 50 mg Available Doses: 25, 50, 100 mg	Onset: 30–60 min Duration: up to 12 h	24‐week study: Improved erections in 56% (25 mg), 77% (50 mg) and 84% (100 mg) vs. 25% for placebo [1]
Vardenafil *2003*	Sulfonamide derivative 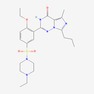	Structurally similar to sildenafil but with an ethyl (vs. methyl) substitution on the piperazine ring and a different orientation; differences in the heterocyclic ring system (mimicking the cGMP purine ring) result in similar binding interactions with PDE5. Shares Q‐pocket/H‐pocket binding with sildenafil; 20‐fold higher PDE5 affinity than sildenafil in vitro	Starting Dose: 10 mg Available Doses: 5, 10, 20 mg	Onset: 15–30 min Duration: Up to 12 h	12‐week study: Improved erections in 66% (5 mg), 76% (10 mg) and 80% (20 mg) vs. 30% for placebo [2]
Tadalafil *2003*	β‐Carboline derivative 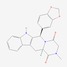	Distinct heterocyclic structure, unique hydrophobic interactions; binds PDE5 with distinct interactions in the Q‐pocket and does not interact with the L region; exhibits 200–600‐fold greater selectivity for PDE5 over PDE6 compared to sildenafil and vardenafil	Starting Dose: 10 mg Available Doses: 10, 20 mg Daily Dose: 5 mg. Available in a daily dosing option	Onset: approx. 30 min Duration: Up to 36 h (longest duration of action)	12‐week study: Improved erections in 67% (10 mg) and 81% (20 mg) vs. 35% for placebo [3]
Avanafil *2012*	Pyrimidine derivative 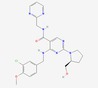	Optimised for rapid absorption and rapid dissociation kinetics for faster onset. Structurally distinct from earlier PDE5Is; inhibits PDE5 to enhance NO‐mediated effects; may exhibit greater selectivity for PDE5 compared to earlier agents (clinical significance unclear)	Starting Dose: 100 mg Available Doses: 50, 100, 200 mg	Onset: As early as 15 min; typically 15–30 min pre‐activity Duration: Up to 6 h	In general ED populations, successful sexual attempts were reported in 47% (50 mg), 58% (100 mg) and 59% (200 mg) vs. 28% for placebo [4]

Abbreviations: cGMP, cyclic guanosine monophosphate; DM, diabetes mellitus; ED, erectile dysfunction; NO, nitric oxide; PDE5, phosphodiesterase type 5; PDE5Is, phosphodiesterase type 5 inhibitors; PDE6, phosphodiesterase type 6.

^a^ Two‐dimensional structures of Sildenafil, Vardenafil, Tadalafil, and Avanafil were obtained from PubChem (Sildenafil: CID 135398744; Vardenafil: CID 135400189; Tadalafil: CID 110635; Avanafil: CID 9869929), National Center for Biotechnology Information. Retrieved from [5].

PDE5Is are generally well‐tolerated for the treatment of ED; however, an absolute contraindication is the concomitant use of any form of organic nitrate or NO donor, including recreational use of amyl nitrite (“poppers”). Concomitant use results in cGMP accumulation and unpredictable, potentially severe hypotension [172].

##### 2.7.2.2 Prostaglandin E1 Agonists

Alprostadil, a synthetic prostaglandin E1 (PGE1) agonist, can be administered via two intraurethral formulations. The first is a topical cream containing a permeation enhancer to facilitate alprostadil absorption (200 and 300 μg) through the urethral meatus [173]. The second is an intraurethral suppository (Medicated Urethral System for Erection, MUSE) containing alprostadil (125–1000 μg) [174]. Common side effects include penile erythema, burning and pain, which typically resolve within 2 h of application.

Alprostadil (Caverject, Edex/Viridal) was the first drug approved for intracavernosal treatment of ED and is most effective as monotherapy at doses ranging from 5 to 40 μg [175]. ICI of vasoactive agents, such as papaverine and PGE1, has been a significant advancement in the treatment of ED and can also be used diagnostically. ICI is an effective local therapy for ED, allowing for individualised treatment plans tailored to patient‐specific needs and conditions. Combining different vasoactive agents and adjusting injection doses can improve treatment outcomes and reduce the risk of complications.

Erection typically occurs within 5–15 min, and the duration is dose‐dependent, although there is significant inter‐individual variability. An in‐office training programme is required to ensure patients learn proper injection technique. Efficacy rates for intracavernosal alprostadil exceed 70% in the general ED population, as well as in specific subgroups (e.g., men with DM or CVD). Reported satisfaction rates range from 87% to 93.5% in patients and 86% to 90.3% in partners [176]. Complications of intracavernosal alprostadil include penile pain (reported by 50% of patients but occurring after only 11% of injections), prolonged erections (5%), priapism (1%) and penile fibrosis (2%) [177].

With the advent of PDE5Is, the clinical use of ICI has decreased due to its higher dropout rate and potential for complications such as priapism, ecchymoses, haematoma and penile fibrosis. Currently, ICI combined with penile Doppler ultrasound is primarily used in the diagnostic evaluation of ED, particularly for assessing penile haemodynamics [178].

##### 2.7.2.3 Hormonal Therapy

Testosterone therapy (intramuscular, transdermal, or oral formulations) may be considered when clinically indicated for men with low or low‐normal testosterone levels and coexisting issues with sexual desire, erectile function, or overall sexual satisfaction [179].

Importantly, androgen replacement therapy (ART) combined with PDE5Is can be effective in treating ED, with some evidence suggesting sustained improvements in erectile function even after discontinuation of the PDE5I [178]. ART can restore serum testosterone to normal levels and improve sexual desire in men with hypogonadism. Studies have also shown improvements in mood and depression with ART. In older men (over 65 years) with low testosterone, one study reported increased frequency of sexual activity and improved sexual desire after 1 year of ART.

#### 2.7.3 Low‐Intensity Extracorporeal Shock Wave Therapy (Li‐SWT)

Low‐intensity extracorporeal shock wave therapy (Li‐SWT) involves the application of low‐energy acoustic waves (energy density < 0.1 mJ/mm^2^) to the penile tissue. As a non‐invasive treatment, Li‐SWT targets the affected tissue by transmitting acoustic waves through the tissue. It has been increasingly investigated as a treatment for vasculogenic ED over the past decade, and it is presented as a potential curative option, which is a highly desirable outcome for the majority of men with ED [180].

Li‐SWT is proposed to stimulate the expression of eNOS, VEGF and other angiogenic factors within the corpora cavernosa. This, in turn, may lead to vasodilation, neovascularisation, increased blood flow and improved erectile function. It has also been shown to be a safe and effective treatment option for patients who have an inadequate response to PDE5Is. Furthermore, some evidence suggests that Li‐SWT may reverse PDE5I resistance, with over 50% of such patients achieving sufficient erectile rigidity after treatment [178].

Overall, while several single‐arm trials have shown a beneficial effect of Li‐SWT on patient‐reported erectile function (EF), data from prospective, randomised, controlled trials (RCTs) are conflicting. Many questions remain unanswered due to the heterogeneity in study designs, including variations in shockwave generators, energy levels, treatment protocols and patient populations [181].

#### 2.7.4 Platelet‐Rich Plasma (PRP)

Platelet‐rich plasma (PRP) is an autologous blood product with a platelet concentration above baseline levels. It is obtained by centrifuging a patient's whole blood, separating it into three components: red blood cells, platelet‐poor plasma (PPP) and PRP. The platelet concentration in PRP can be up to five times higher than in whole blood. PRP has garnered significant clinical interest due to its high concentration of platelets, which contain alpha granules. These small secretory granules fuse with the platelet plasma membrane, releasing hundreds of bioactive proteins and growth factors [182].

The regenerative effects of PRP are attributed to its high concentration of platelets, which contain numerous growth factors, including VEGF, epidermal growth factor (EGF), insulin‐like growth factor‐1 (IGF‐1), platelet‐derived growth factor (PDGF) and fibroblast growth factor (FGF). These growth factors are believed to promote angiogenesis and stem cell recruitment, potentially contributing to tissue repair and regeneration in patients with ED [183].

The results were confirmed with a prospective, randomised, double‐blind, placebo‐controlled study that was conducted on 109 patients (aged 45–65 years) with mild‐to‐moderate ED who had discontinued all other ED treatments. At 1, 3 and 6 months post‐PRP injection, patients in the PRP group showed significant improvements compared to the placebo group, as measured by questionnaires such as the IIEF [184].

#### 2.7.5 Vacuum Erection Devices

A vacuum erection device (VED) is a mechanical instrument that creates a vacuum around the penis, drawing blood into the corpora cavernosa and inducing an erection. A constriction ring placed at the base of the penis helps maintain the erection. They are primarily used to treat organic ED and are generally considered safe and effective. While VEDs can be used as an alternative in patients with inadequate response to PDE5Is, their use has also been explored for penile rehabilitation [178]. Long‐term adherence to VED therapy is variable, with studies reporting usage rates of 50%–64% after 2 years [185]. The most common adverse events include penile pain, inability to ejaculate, petechiae, bruising and numbness [186]. They are contraindicated in patients with bleeding disorders or those receiving anticoagulant therapy [187].

#### 2.7.6 Penile Prostheses

Penile prosthesis implantation (PPI) is generally considered a third‐line treatment for ED. Because PPI can cause irreversible changes to the corpora cavernosa, it is typically considered when oral PDE5Is, ICIs and VEDs are ineffective or contraindicated. PPI is indicated in patients who: (i) are not candidates for, or prefer not to use, other pharmacotherapies, or desire a permanent solution; and (ii) have not responded to other treatment modalities [188].

The two main classes of penile implants are inflatable (two‐ and three‐piece) and semi‐rigid (malleable, mechanical and soft flexible) devices [189]. The two primary surgical approaches for PPI are penoscrotal and infrapubic. A systematic review comparing satisfaction and complication rates found no significant advantage between the two approaches. Therefore, it is recommended that surgeons be proficient in both techniques and able to tailor the incision strategy to individual patient anatomy and complexity [190].

The three‐piece inflatable prosthesis is currently the most commonly implanted device and generally provides the highest level of patient satisfaction. It allows for manual control over penile rigidity and simulates a natural erection more closely than other implant types. While it offers advantages, PPI is costly, invasive and carries a risk of complications, including prosthetic infection, pump migration, autoinflation and the need for revision surgery [178].

#### 2.7.7 Emerging and Experimental Therapies

##### 2.7.7.1 Gene Therapy

Gene therapy for ED involves delivering genes that encode proteins with impaired function in the penile tissue of men. Replacing or supplementing these proteins may improve erectile function. Experimental animal models have demonstrated promising results with gene therapy. While human studies are limited, they may also demonstrate efficacy. However, gene therapy faces significant hurdles, including regulatory approval and public acceptance, which may delay its widespread clinical implementation.

Gene therapy for ED may offer a valuable therapeutic option for patients with conditions such as severe CVD, those taking nitrates, individuals with DM, obese patients and men who have undergone radical prostatectomy. It has several intrinsic advantages: (i) the penis is easily accessible, allowing for direct intracavernosal administration of the therapeutic gene, minimising systemic exposure; (ii) the relatively low turnover rate of cavernosal smooth muscle cells may allow for long‐term expression of the therapeutic gene; (iii) gap junctions between cavernosal smooth muscle cells may facilitate gene transfer even with relatively low transfection efficiency, due to the resulting syncytial network; and (iv) gene therapy allows for targeted alteration of specific molecular pathways involved in the erectile process, potentially addressing disease‐specific defects and improving erectile response [191].

Gene‐based therapy has been proposed as one of the potential new therapies for PDE5‐resistant ED. Genes involved in NO synthesis, such as NO synthase (NOS), have been tested for the potential gene therapies for ED. On the other hand, genes encoding different types of neurotrophic factors have been used for the neurogenic type of ED induced by DM or cavernous nerve injury. Additionally, K+ channel genes, which enhance cavernous smooth muscle relaxation, have also been tested as another category of genes for ED gene therapy [192].

Adenoviral vector‐mediated overexpression of eNOS has been examined in aged or diabetic rats. It was observed that eNOS transgene expression and cGMP level in cavernous tissues increased when recombinant adenovirus containing the eNOS gene was injected into the corpus cavernosum of aged rats [192]. Other gene therapies that are targeting nitrergic mechanisms are protein inhibitors of NOS, anti‐arginase and cGMP‐dependent protein kinase G1 [192].

Both non‐viral and viral delivery vehicles have been used to deliver genes to the penis and other tissues. A major advantage of the non‐viral delivery systems has been the low immunogenicity of this approach compared to viral vectors. On the other hand, viral vectors are more efficient at delivering their genetic material to the target cell [192].

##### 2.7.7.2 Intracavernosal Administration of Various Cell Lines

Stem cell therapy research holds significant promise for treating ED by addressing underlying issues such as endothelial dysfunction, smooth muscle atrophy and nerve damage. This potential is based on advancements in regenerative medicine and the ability of stem cells to regenerate damaged cavernosal nerves and vasculature in diabetes. The therapeutic effects of mesenchymal stem cells (MSCs) are thought to arise from multiple mechanisms. One proposed mechanism is the direct replacement of damaged endothelial or neuronal cells. Another hypothesis is that MSCs exert paracrine effects, modulating key mediators of erectile function, including calcium and NO signalling. Furthermore, MSCs possess anti‐apoptotic and anti‐fibrotic properties, which may contribute to their therapeutic benefits [193].

###### 2.7.7.2.1 Adipose Tissue Derived Stem Cells (ADSCs)

Adipose tissue is a readily accessible and abundant source of MSCs. ADSCs are multipotent cells that exhibit anti‐apoptotic, pro‐angiogenic and immunomodulatory properties. The paracrine effects of ADSCs, mediated by the release of various bioactive molecules, have received significant attention. These secreted factors include VEGF, brain‐derived neurotrophic factor (BDNF) and other factors that may improve erectile function in the context of DM [193].

###### 2.7.7.2.2 Bone Marrow‐Derived Stem Cells (BMSCs)

Bone marrow‐derived stem cells (BMSCs) are another source of MSCs used in therapeutic applications. MicroRNAs (miRNAs) play a significant role in regulating BMSC function. Specifically, miRNAs are known to be crucial regulators of stem cell self‐renewal and differentiation into various cell types, including smooth muscle cells, which are essential for maintaining erectile function [193].

###### 2.7.7.2.3 Umbilical Cord Mesenchymal Stem Cells (UC‐MSCs)

Human umbilical cord‐derived mesenchymal stem cells (UC‐MSCs) are multipotent cells. UC‐MSCs can be readily harvested in large quantities from umbilical cord tissue. Compared to BMSCs and ADSCs, UC‐MSCs have shown promising results in preclinical and some clinical studies for ED treatment. Key advantages of UC‐MSCs include a non‐invasive collection method, minimising donor site morbidity; high proliferative capacity, allowing for expansion in vitro even after multiple passages; and a relatively low risk of tumourigenicity, potentially due to their well‐characterised gene expression profile [193].

#### 2.7.8 Limitations and Considerations in Treatment Approaches

Ethical considerations are critical in the development and clinical use of stem cell and gene therapies for ED. The promise of autologous restorative treatments, coupled with lengthy regulatory procedures, may drive both providers and patients towards unproven interventions with unclear benefits and risks. The advancement of cellular therapies for ED depends on large, randomised, placebo‐controlled trials. However, there is currently a lack of such well‐designed studies in this area. To address this, coordinated multicentre trials with adequate statistical power are required. Research must rigorously define safety, efficacy, optimal dosing and delivery methods. Although preclinical studies in animal models have shown encouraging results, successful translation into robust human trials remains essential [194].

## 3 Conclusions

Diabetic ED represents a significant and prevalent clinical challenge, substantially diminishing the quality of life for affected men. This review highlighted the complex, multifactorial pathophysiology of diabetic ED, encompassing a network of interacting mechanisms. Endothelial dysfunction, accelerated atherosclerosis and both autonomic and peripheral neuropathy are central to the vascular and neural impairments that characterise the condition. These processes are further exacerbated by structural alterations in penile tissue, hormonal imbalances and psychological factors, all operating within the context of chronic hyperglycaemia and often co‐occurring with other DM‐related complications and comorbidities.

Current management of diabetic ED is multimodal, relying on lifestyle interventions, psychosexual counselling and a range of pharmacological options, primarily PDE5Is. While PDE5Is are effective for the majority of patients, treatment response is variable and some individuals experience inadequate or no benefit. This variability underscores the need for a more personalised approach to treatment. Emerging therapies, such as intracavernosal stem cell injections and gene therapy, hold promise for addressing the underlying pathophysiology but require further investigation in large‐scale, randomised controlled trials.

Pharmacogenomics offers a crucial avenue for advancing personalised treatment. This review has highlighted the associations between specific genetic polymorphisms (e.g., in *NOS3*, *ACE*, *AR*, *VEGF* and *ARG1*) and both ED risk and, potentially, treatment response. Integrating these genetic insights with clinical and pathophysiological data holds the key to predicting individual responses to different therapies. Future research should prioritise large, well‐designed pharmacogenetic studies to identify robust genetic biomarkers that can guide treatment selection. Ultimately, a deeper understanding of the interplay between genetic predisposition, pathophysiological mechanisms and treatment response will pave the way for more effective and individualised management strategies for diabetic ED, improving outcomes and enhancing the quality of life for men with this condition.

## Author Contributions

Conceptualisation, B.H., G.T. and J.K.; methodology, B.H., G.T. and J.K.; writing – original draft, B.H., G.T. and J.K.; writing – review and editing, J.K.; visualisation, G.T.; supervision, J.K. and A.J. All authors have read and agreed to the published version of the manuscript.

## Conflicts of Interest

The authors declare no conflicts of interest.

## Data Availability

The data that support the findings of this study are available from the corresponding author upon reasonable request.
